# Label Accuracy in Electronic Health Records and Its Impact on Machine Learning Models for Early Prediction of Gestational Diabetes: 3-Step Retrospective Validation Study

**DOI:** 10.2196/72938

**Published:** 2025-08-21

**Authors:** Mark Germaine, Amy C O'Higgins, Brendan Egan, Graham Healy

**Affiliations:** 1 School of Computing, Dublin City University, Glasnevin, Dublin, D09 V209, Ireland, 353 1 700 8803; 2 School of Health and Human Performance, Dublin City University, Dublin, Ireland; 3 UCD (University College Dublin) Centre for Human Reproduction, Coombe Women & Infants University Hospital, Dublin, Ireland

**Keywords:** electronic health records, gestational diabetes, label noise, pregnancy, validation, machine learning

## Abstract

**Background:**

Several studies have used electronic health records (EHRs) to build machine learning models predicting the likelihood of developing gestational diabetes mellitus (GDM) later in pregnancy, but none have described validation of the GDM “label” within the EHRs.

**Objective:**

This study examines the accuracy of GDM diagnoses in EHRs compared with a clinical team database (CTD) and their impact on machine learning models.

**Methods:**

EHRs from 2018 to 2022 were validated against CTD data to identify true positives (TP), false positives (FP), true negatives (TN), and false negatives (FN). Logistic regression models were trained and tested using both EHR and validated labels, whereafter simulated label noise was introduced to increase FP and FN rates. Model performance was assessed using the area under the receiver operating characteristic curve (ROC AUC) and average precision (AP).

**Results:**

Among 3952 patients, 3388 (85.7%) were correctly identified with GDM in both databases, while 564 cases lacked a GDM label in EHRs, and 771 were missing a corresponding CTD label. Overall, 32,928 (87.5%) of cases were TN, 3388 (9%) TP, 771 (2%) FP, and 564 (1.5%) FN. The model trained and tested with validated labels achieved an ROC AUC of 0.817 and an AP of 0.450, whereas the same model tested using EHR labels achieved 0.814 and 0.395, respectively. Increased label noise during training led to gradual declines in ROC AUC and AP, while noise in the test set, especially elevated FP rates, resulted in marked performance drops.

**Conclusions:**

Discrepancies between EHR and CTD diagnoses had a limited impact on model training but significantly affected performance evaluation when present in the test set, emphasizing the importance of accurate data validation.

## Introduction

Electronic health records (EHRs) are an important source of real-world data, offering detailed, longitudinal patient information historically stored in medical charts, and forming the basis of real-world evidence [1,2]. Together with advancements in artificial intelligence and machine learning (ML), EHRs are increasingly being used to develop models that improve the prediction of health and disease outcomes [3].

Integration of EHRs into clinical research offers numerous opportunities for advancing health care delivery and patient outcomes. However, EHR data is often stored in unstructured formats such as free text, requiring information extraction algorithms to enable ML applications [4]. This extraction process can introduce data quality concerns due to various issues such as data entry errors and cut-and-paste errors [5]. The quality and consistency of EHR data are particularly critical when the target variable, that is, the variable being predicted, is used in ML models.

Inaccuracies in EHRs present challenges for developing and applying ML algorithms in health care, primarily due to the dependency on data quality and accuracy of target labels [6]. This “label noise,” which refers to inaccuracies or inconsistencies in the data labels (eg, diagnosis codes) extracted from EHRs, can significantly impact model performance by introducing errors in the target variable, leading to potentially misleading conclusions [7]. Training ML models on unvalidated EHRs may lead to systematic errors in the model output with the potential for the model to miss, underestimate, or overestimate clinically significant relationships [8,9].

Accurate diagnosis and recording of gestational diabetes mellitus (GDM) in EHRs is important not only for effective patient management but also for informing public health strategies and economic forecasting in national health care planning [10,11]. EHRs are often used to train ML approaches that support clinical decision-making and care pathways that improve pregnancy outcomes [12]. However, the utility of EHRs remains a concern due to potential discrepancies in data recording practices [8]. When using ML in GDM prediction [13], the accuracy of input data is paramount because inaccuracies can lead to flawed prediction models and ineffective or adverse clinical decisions [14].

Several studies have used EHRs to build ML models predicting the likelihood of developing GDM later in pregnancy [15], but none have described validation of the GDM “label” within the EHRs. This study has 3 primary aims: first, to assess the accuracy of reporting of GDM diagnoses in EHRs by comparing them to a database maintained in real-time by the hospital’s clinical team; second, to evaluate how discrepancies in GDM reporting impact ML models; and third, to examine ML model performance using varying levels of simulated label noise in the dataset. By identifying discrepancies between these data sources, we aim to highlight the importance of data validation for advancing digital health and ML-driven health care.

## Methods

### Study Design

A retrospective validation design was used to assess the accuracy of GDM diagnoses recorded in the EHRs of a national maternity hospital (The Coombe Hospital, Dublin). We matched patient ID between the EHR system and a reference standard established by a real-time clinical team database (CTD) of those formally diagnosed with and managed for GDM, which served as a ground truth. This approach allowed for direct comparison between the recorded GDM status in the EHRs and the validated GDM status from the CTD, enabling identification of true positives (TP), false positives (FP), true negatives (TN), and false negatives (FN) in the EHRs. Further, the effect of label noise on ML model performance in predicting the development of GDM (binary classification) was evaluated by first examining its impact in our current EHR dataset, and then second, simulating progressively increasing levels of label noise to understand its effect on ML model performance both in terms of training and testing.

### Data Source and Validation

The EHR system serves as the repository for patient medical histories, including diagnoses, family history, and outcomes for pregnant women receiving care at the institution. The data are collected routinely from all women by trained midwives using standardized questions and are then computerized onto the electronic system of the hospital, “Euroking K2.” EHRs were collected from 2018 to 2022 and consisted of over 35,000 pregnancies during this time. The dataset from the CTD spanned from 2018 to 2022; thus, the timeframe for this analysis spanned from 2018 to 2022 (inclusive). Women aged 18 years or older with complete information on GDM status were included in the analysis. Pregnancies with missing or incomplete data for critical variables, women without a recorded GDM status, and pregnancies with pre-existing diabetes were excluded. ML models were trained and tested on pregnancies with complete EHR data up to the 12th week of gestation.

GDM diagnoses were extracted from the EHRs based on information recorded in a column titled “medical problems during pregnancy.” When this column contained the entry “diabetes developed during pregnancy,” the patient was coded as having GDM in a newly created column designated for this study’s analysis, referred to hereafter as “EHR-GDM.” Patient records not meeting this criterion were coded as not having GDM. The legacy EHR has a single structured problem field; it does not store *ICD* (*International Statistical Classification of Diseases and Related Health Problems*) or SNOMED (Systematized Nomenclature of Medicine) codes. GDM is recorded exclusively by selecting “diabetes developed during pregnancy” from that field’s drop-down list. No alternative structured or coded location exists.

Patient IDs from the EHRs were then matched with a separate database maintained in real-time by the clinical team responsible for diabetes care, with patient details entered each day upon confirmation from the hospital laboratory of a diagnosis of GDM from an oral glucose tolerance test following the IADPSG (International Association of the Diabetes and Pregnancy Study Groups) guidelines. The CTD was considered the definitive ground truth for GDM diagnoses, given its real-time, clinician-entered, laboratory-confirmed data recording process. This matching process produced a merged dataset for validating EHR-recorded GDM diagnoses against the CTD database, leading to the creation of 2 comparison columns: “EHR-GDM” for EHR-identified cases of GDM and “CTD-GDM” for cases of GDM recorded by the CTD.

The validation process involved comparing the GDM diagnosis status in the EHR (“EHR-GDM”) with that in the CTD (“CTD-GDM”) to examine the agreement between the 2 datasets. This comparison allowed for the identification of TPs (positive in EHRs and present in CTD), FPs (positive in EHRs and not present in CTD), TNs (negative in EHRs and not present in CTD), and FNs (negative in EHRs and present in CTD), and thereby enabled evaluation of the accuracy of the reporting of GDM diagnosis in the EHRs. An additional column, validated gestational diabetes mellitus label (only in cases where electronic health record and clinical team database labels matched; VAL-GDM), was created indicating a positive or negative diagnosis of GDM for cases where the EHR-GDM and CTD-GDM labels matched, that is, for TPs and TNs, excluding records with FPs and FNs. Thus, for the purpose of the following stage of ML modeling, only records that matched between EHR-GDM and CTD-GDM were used, reducing the risk of bias from either dataset. The TPR, FPR, TNR, and FNR were calculated for the dataset [16].

### Evaluation of Label Noise on ML Modeling

To evaluate the impact of label noise on the performance of ML models in predicting GDM, we used logistic regression (LR), where the dataset was split into 70% training and 30% test sets to ensure robust evaluation. Default model hyperparameters were used, as the primary objective was to compare performance across different training datasets rather than optimizing hyperparameter settings. The training and testing data comprised EHR data that was available during the first booking visit, typically the 12th week of gestation, and included 79 training features. The target label was GDM. The dataset contained both categorical and numerical features. Categorical features were processed using OneHotEncoder with the “first” category dropped, and numerical features were standardized using StandardScaler. While the goal of this paper is not to produce an end point AI model, a self-assessment checklist for reporting was followed to ensure that adequate information about the ML model was present [17].

We trained 2 ML models: one with the EHR-GDM labels and the other with the VAL-GDM labels. Both models were evaluated using the same test set, which used VAL-GDM labels, to facilitate a direct comparison of the effects of label noise during training on a consistent test set. The year 2020 was excluded from these analyses due to emerging research demonstrating reduced detection of diseases during this period [18], something that we confirm in our results below. By using both the “raw” and “validated” datasets, the study aimed to demonstrate the impact of label noise on model performance in the prediction of GDM, providing insights into the importance of accurate label validation in developing reliable predictive models using ML. In addition to the LR model, we replicated this process with other ML models to ensure any effects were not model specific. External validation datasets were sought, but this was not successful, as documented [19].

Additionally, varying levels of label noise were introduced to determine the threshold at which label noise significantly affects model performance. This simulation was performed by progressively increasing the number of FPs and FNs in the VAL-GDM training set from 0% to 90%, that is, changing a percentage of the positive labels to negative labels (creating FNs) and changing a percentage of negative labels to positive labels (creating FPs). This approach resulted in the training of 100 different models. Next, in a separate analysis, we applied this progressive noise insertion to the VAL-GDM test set to specifically assess the impact of test set label noise on model evaluation, that is, evaluating these test sets using a model trained with the “clean” VAL-GDM labels. For reproducibility, the code used to perform the label noise simulation is made available in the Simulated Label Noise section.

### Statistical Analysis

The validation findings were quantitatively assessed using accuracy, precision, recall, *F*_1_-score, and overall agreement measured by Cohen κ, between the EHR-recorded GDM (EHR-GDM) and the CTD (CTD-GDM) diagnoses. The performance of the LR ML models was evaluated using the area under the receiver operating characteristic curve (ROC AUC) and the average precision (AP) score. Additionally, the calibration of the model’s predictions will be examined visually by calibration curves and quantitatively by the slope and intercept. The statistical and ML analyses were performed using Python (version 3.8.8; Python Software Foundation) with libraries including NumPy 1.23.5, pandas 1.2.4, and scikit-learn 1.2.1.

### Ethical Considerations

Ethical approval was granted by The Coombe Hospital Research Ethics Committee (Study No. 06–2023; Exploring the Utility of Machine Learning for the Classification of Gestational Diabetes Risk During the First Antenatal Visit).

## Results

### Population Characteristics

The dataset comprised 37,651 EHRs from 31,100 unique patients. The mean patient age was 32 (SD 5) years, and BMI was 26.2 (SD 5.5) kg/m^2^, with 20.7% exhibiting a BMI greater than 30.0 kg/m^2^. The prevalence of GDM according to the EHRs was 11%, whereas the prevalence according to the CTD was 10.5%. Patient characteristics for the most important features in the ML models are presented in Table 1.

**Table 1.** Patient characteristics for the most important features in the machine learning models, according to the validated dataset (n=27,561). The validated dataset represents a dataset where both the EHRs^a^ andCTDs^b^ agree.

Characteristics	Values
Age (years), mean (SD)	32 (5)
BMI (kg/m^2^), mean (SD)	26.2 (5.3)
Systolic blood pressure (mm Hg), mean (SD)	111 (11)
Diastolic blood pressure (mm Hg), mean (SD)	67 (8)
Parity, mean (SD)	0.9 (1.1)
Ethnic origin, n (%)	
Caucasian	24,180 (87.8)
South East Asian	1360 (4.9)
Black African	554 (2.0)
Asian	489 (1.8)
Middle Eastern	154 (0.6)
Latin American	26 (0.1)
Mixed	10 (0.1)
Other	788 (3)
Occupation skill level (ISCO^c^), n (%)	
Level 0 (unemployed)	5254 (19)
Level 1 (elementary occupations)	369 (1.3)
Level 2 (clerical and service)	4404 (15.9)
Level 3 (technicians and associates)	2389 (8.6)
Level 4 (professionals and managers)	15,145 (55.1)
Family history of diabetes mellitus, n (%)	6407 (23.3)
History of GDM^d^, n (%)	1078 (3.9)
Other endocrine problems, n (%)	5854 (21.4)
Prevalence of GDM, n (%)	3188 (11.7)

a EHR: electronic health record.

b CTD: clinical team database.

c ISCO: International Standard Classification of Occupations.

d GDM: gestational diabetes mellitus.

### Diagnosis Discrepancies

Of 3952 patients with matching IDs in both databases, 3388 were correctly identified with GDM in both EHR-GDM and CTD-GDM (9% TP and 85.7% true positive rate [TPR]), while 564 lacked a corresponding GDM label in EHR-GDM (1.5% FN and 14.3% false negative rate [FNR]). Additionally, 771 patients were incorrectly identified with GDM in EHR-GDM without matching IDs in CTD-GDM (2% FP and 2.3% false positive rate [FPR]). In EHRs, there were 32,928 (87.5%) TN cases (97.7% true negative rate [TNR]). The accuracy, precision, *F*_1_-score, and Cohen κ are reported in Table 2.

**Table 2.** Performance metrics for the comparison of GDM^a^ diagnoses in EHRs^b^ with the real-time CTD^c^.

Year	Cohen κ	Accuracy	Precision	Recall	*F*_1_-score
All years	0.82	0.96	0.81	0.86	0.84
2018	0.80	0.96	0.78	0.86	0.82
2019	0.82	0.96	0.86	0.82	0.84
2020	0.77	0.96	0.70	0.90	0.79
2021	0.86	0.97	0.89	0.87	0.88
2022	0.82	0.97	0.82	0.86	0.84
All minus 2020	0.82	0.97	0.84	0.85	0.84

a GDM: gestational diabetes mellitus.

b EHR: electronic health record.

c CTD: clinical team database.

### Yearly Data Comparison

**Figure 1.**
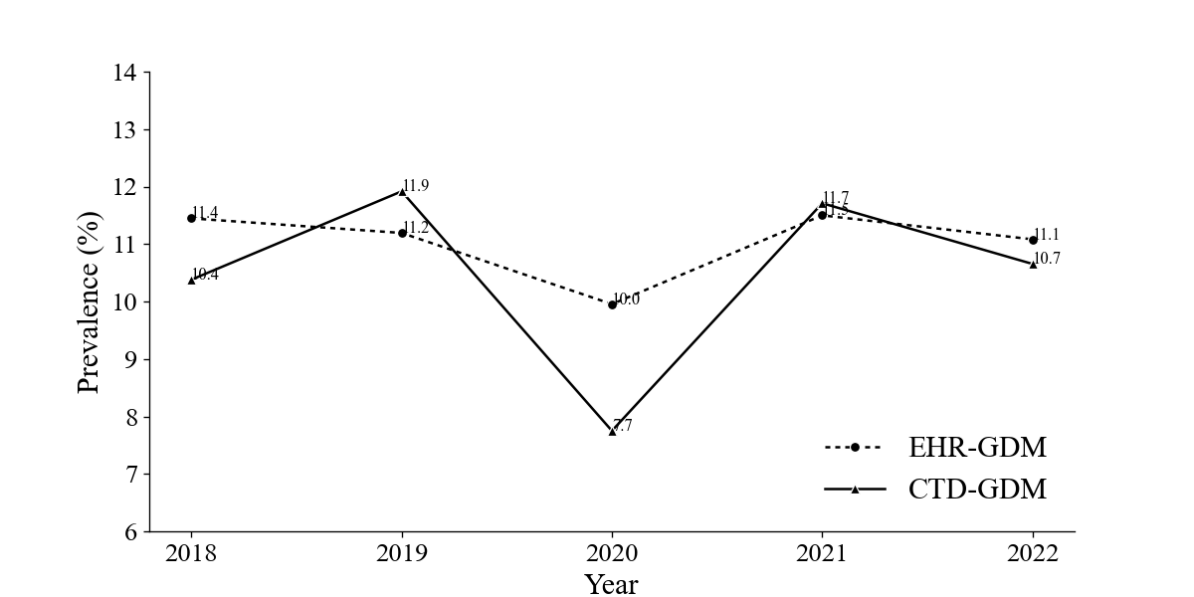
Comparison of prevalence rates of EHR-GDM data and CTD-GDM data from 2018 to 2022. The solid line represents the CTD data, and the dashed line represents the EHR data. CTD: clinical team database; CTD-GDM: gestational diabetes mellitus label as recorded in the clinical team database (reference standard label); EHR: electronic health record; EHR-GDM: gestational diabetes mellitus label as recorded in the EHR.

Ninety-eight patients identified in CTD lacked corresponding entries in EHRs. In total, 67 (68%) of these discrepancies were observed in 2020. Furthermore, GDM prevalence for both EHRs and CTD datasets revealed a notable reduction in 2020 (recorded at 10% in EHRs and 7.7% in CTD), indicating a deviation from the trend observed in other years (Figure 1). These discrepancies align with COVID-19–related disruptions to screening practices within the hospital between March 2020 and September 2020.

### Label Noise in EHRs

**Figure 2.**
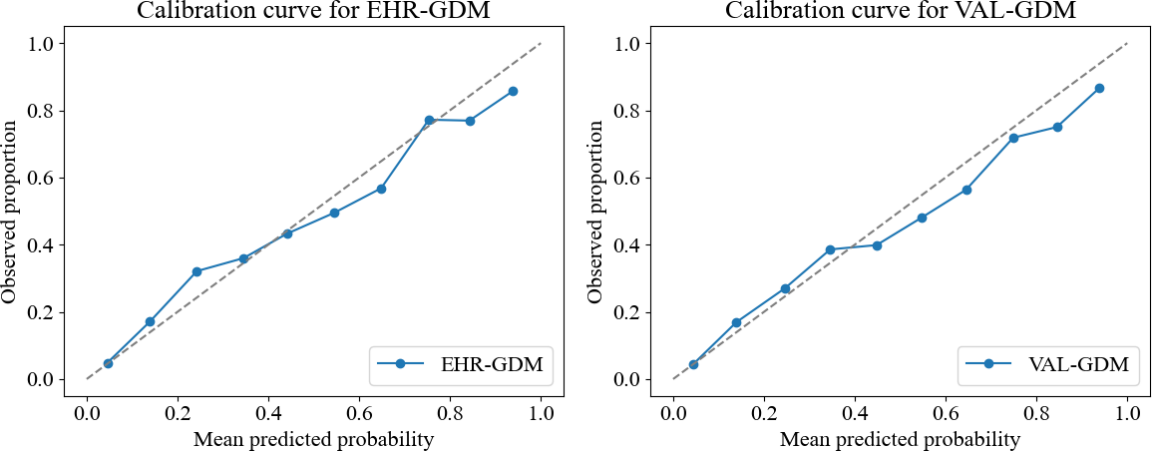
(Left) Calibration curve for the EHR (electronic health record)-GDM model, which was trained on EHR-GDM labels. (Right) Calibration curve for the VAL-GDM model, which was trained on the subset of cases where electronic health record and clinical team database labels agree. Both are evaluated against the identical standard reference labels. EHR-GDM: gestational diabetes mellitus label as recorded in the EHR; GDM: gestational diabetes mellitus; VAL-GDM: validated gestational diabetes mellitus label (only in cases where the EHR and clinical team database labels matched).

**Figure 3.**
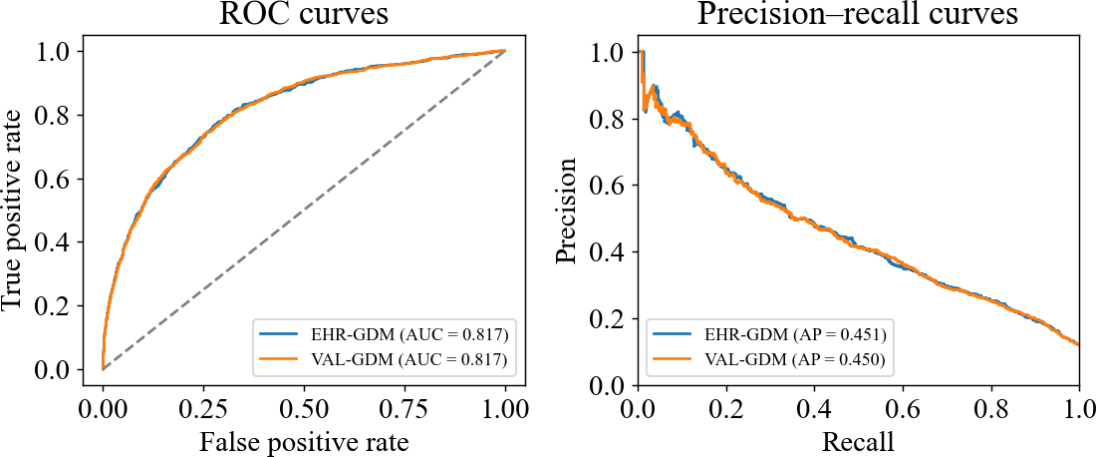
(Left) ROC curves and (right) precision‐recall curves showing the performance of 2 logistic‐regression models for predicting GDM. “EHR-GDM” refers to the model trained on electronic health record (EHR)–GDM labels, and “VAL-GDM” refers to the model trained on the subset of cases where the EHR and clinical team database labels agree. Both models are evaluated against the same reference labels (VAL-GDM). EHR: electronic health record; EHR-GDM: gestational diabetes mellitus label as recorded in the EHR; GDM: gestational diabetes mellitus; ROC: receiver operating characteristic; VAL-GDM: validated gestational diabetes mellitus label (only in cases where EHR and clinical team database labels matched).

The performance of LR models trained using the raw (EHR-GDM) and validated (VAL-GDM) labels was evaluated using a test set with VAL-GDM labels only. The model trained using the EHR-GDM labels achieved an ROC AUC of 0.817 (95% CI 0.802‐0.833) and an AP score of 0.451. The calibration curve is shown in Figure 2, with an intercept of 0.093 and a slope of 0.984. In comparison, the model trained using the VAL-GDM labels showed an ROC AUC of 0.817 (95% CI 0.803‐0.832) and an AP score of 0.450 (Figure 3), indicating a minor impact of label noise in training the model for this dataset (intercept −0.027 and slope 0.955). However, when the performance of the LR ML model trained using VAL-GDM labels was evaluated on a test set with EHR-GDM labels, an ROC AUC of 0.814 and an AP score of 0.395 was achieved, which demonstrates a greater impact of label noise when it is present in the test set.

In addition to LR, random forest, XGBoost (Extreme Gradient Boosting), and an Explainable Boosting Machine were assessed to compare their performance and robustness to label noise. As shown in Table 3, all 3 models achieved performance metrics in a similar range to the LR model, with none of the models demonstrating large changes in evaluation metrics regardless of the validation data used.

**Table 3.** Comparison of ROC AUC^a^ and average precision for machine learning models predicting GDM^b^ trained on the EHR^c^ data and VAL^d^ data, and validated against the VAL data.

Model	ROC AUC		Average precision		Intercept		Slope	
	EHR-GDM	VAL-GDM	EHR-GDM	VAL-GDM	EHR-GDM	VAL-GDM	EHR-GDM	VAL-GDM
Logistic regression	0.817	0.817	0.451	0.450	0.093	−0.027	0.984	0.955
Random forest	0.797	0.801	0.418	0.419	−0.747	−0.618	0.553	0.638
XGBoost^e^	0.780	0.782	0.389	0.393	−0.427	−0.507	0.619	0.608
EBM^f^	0.818	0.816	0.456	0.450	0.078	−0.047	0.975	0.940

a ROC AUC: area under the receiver operating characteristic curve.

b GDM: gestational diabetes mellitus.

c EHR: electronic health record.

d VAL: validated.

e XGBoost: Extreme Gradient Boosting.

f EBM: Explainable Boosting Machine.

### Simulated Label Noise

**Figure 4.**
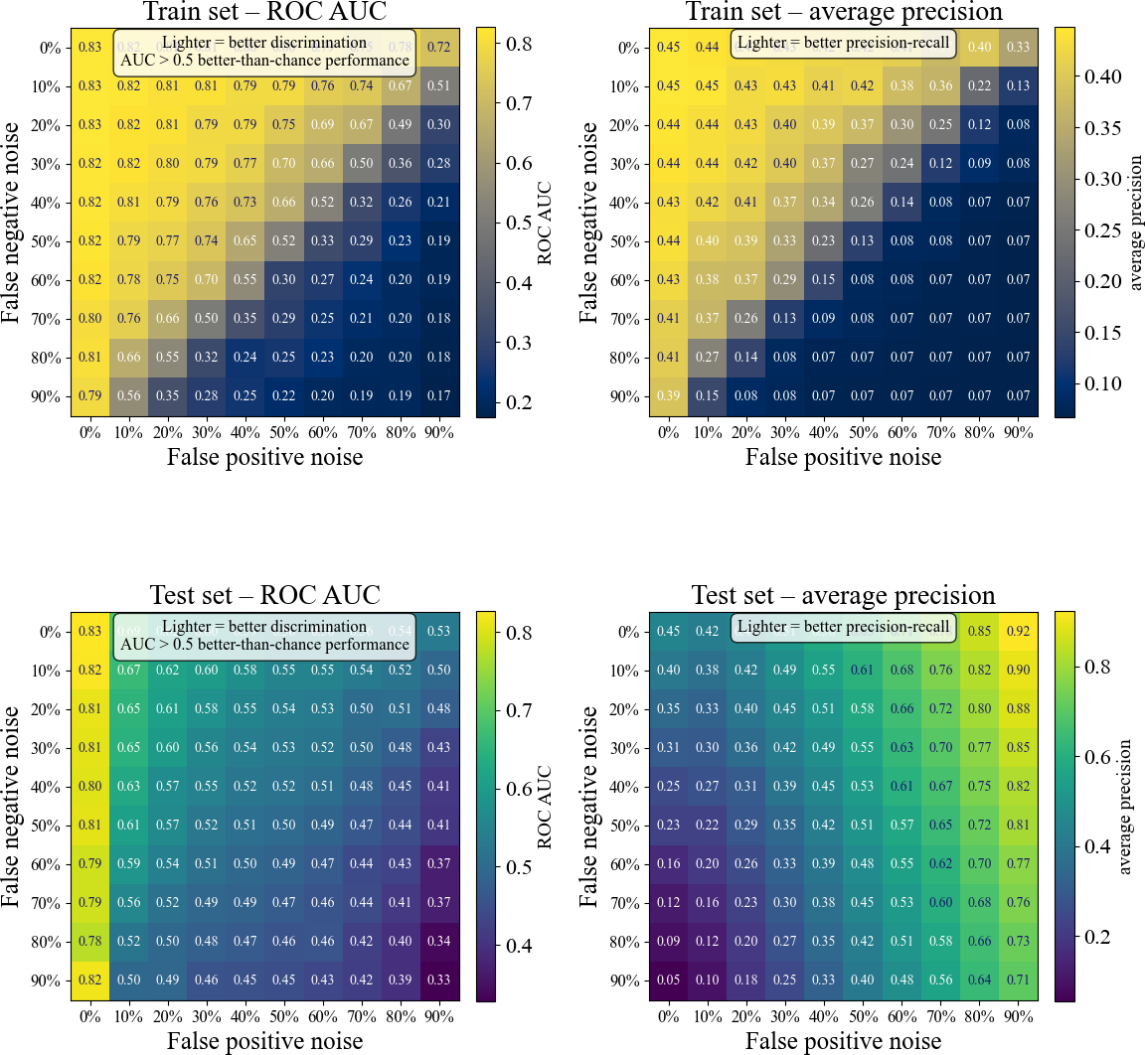
Heatmaps illustrating how gradually introducing random label noise (NAR) degrades model performance. In each panel, the x-axis denotes the percentage of true negatives flipped to false positives, and the y-axis denotes the percentage of true positives flipped to false negatives. In the top-left heatmap, the ROC AUC on the training set is plotted; lighter cells signify stronger discrimination, and values above 0.5 represent performance better than random chance. The top-right heatmap presents the corresponding average precision on the same noisy training data, with lighter colors indicating a more favorable precision-recall trade-off. The bottom-left and bottom-right heatmaps repeat these experiments on the held-out test set, showing ROC AUC and average precision, respectively, under increasing label noise in the test data. The unusual behavior of average precision is discussed in this paper. Each cell is annotated with the exact metric value for that combination of false positive and false negative noise levels. AUC: area under the curve; NAR: noise at random; ROC AUC: area under the receiver operating characteristic curve.

The impact of simulated label noise on model performance was assessed by progressively increasing the number of FNs (FN noise) and FPs (FP noise) in the training set (where 0% noise equates to the original VAL-GDM labels) without modifying the testing set. The results demonstrate a decline in model performance as the level of label noise increases (Figure 4).

Further analysis of noise in the test set showed that model performance metrics, particularly ROC AUC and AP scores, were sensitive to increasing levels of noise, especially FP noise. As the FP rate was increased, the ROC AUC consistently decreased, while the AP score initially decreased before increasing. The introduction of FN into the test set had a less pronounced effect on performance compared to FP, unless both types of noise were combined, which led to a more substantial impact (Figure 4).

## Discussion

This study highlights significant discrepancies between GDM diagnoses recorded in EHRs and those validated by the CTD. Correcting label noise in the training set had a negligible impact on the performance of an LR-based ML model developed from EHRs to predict GDM from early pregnancy data. However, correcting label noise in the test set improved the model’s AP, underscoring the importance of accurate labeling for evaluating model performance accurately. This study also found that increasing label noise in the training set led to a gradual decline in model performance, while increasing FPs in the test set had a particularly strong negative impact on ROC AUC, but counterintuitively increased AP scores. FNs had a less pronounced impact on ROC AUC unless combined with FP, which then caused a decline in model performance.

Approximately 14% (564/3952) of GDM cases were not recorded in the EHRs, while 18.5% (771/4,159) of positive GDM diagnoses in EHRs did not align with CTD records. Overall, there were 32,928 (87.5%) TN, 3388 (9%) TP, 771 (2%) FP, and 564 (1.5%) FN. The FPR (2.3%) remained low in comparison to the FNR (14.3%). Similar discrepancies in accuracy of EHRs have been reported in previous studies within Irish maternal hospitals, though with higher agreement in other contexts, such as miscarriage measurements (k=0.92) [20]. More widely across Europe, wide variations exist in the accuracy of reporting in EHRs as it relates to acute cardiovascular outcomes, with sensitivity reported at <66% for heart failure diagnoses in particular [21]. A key challenge in these studies is the absence of a recommended reference standard for validating EHR data, leading to the use of various data sources [8].

The impact of COVID-19 on screening and diagnostic practices, especially in 2020, manifested in a relative reduction of 31% in GDM diagnoses, that is, 11.2% across 2018, 2019, 2021, and 2022 compared to 7.7% in 2020. These observations align with research indicating reduced diagnosis rates for various medical conditions during the first year of the pandemic [18], and suggest caution is warranted when using EHRs during this year for the purpose of health care modeling. The decrease in recorded GDM cases in 2020 was likely driven by changes in clinical protocols at the onset of the COVID-19 pandemic. The Irish Health Service Executive adopted procedures recommended by the Royal College of Obstetrics and Gynecologists in the United Kingdom, which recommended alternative testing strategies for screening pregnant women for GDM that focused on replacing the 2-h oral glucose tolerance test with other tests of shorter duration [22].

Correcting label noise has been shown to mitigate its adverse effects on model performance, underscoring the importance of “clean” and accurate datasets for training and validating ML algorithms to ensure their efficacy in clinical decision support systems [23]. For example, training a model on a “clean” dataset resulted in an accuracy of 73.6%, whereas with 30% label noise the accuracy fell to 64.1% (−9.5%) [23]. However, the current analysis demonstrated that training an LR model using EHR-GDM labels versus VAL-GDM yielded negligible differences in performance metrics, with ROC AUCs of 0.817 and 0.817, respectively (Figure 3), a performance in line with previous research using EHRs to predict GDM [13,15]. This is presumably due to the low overall representation of FN and FP in the dataset of 3.5% combined, which limited the impact of label noise on the training process.

Previous work has simulated noisy labels with artificial introduction of different levels of label noise (10%, 20%, and 40%) into the training set and demonstrated a gradual decline in the accuracy of all models (mean AUC of all models at 80.3, SD 3.3, 10%; 79.3, SD 3.4, 20%; and 77.6, SD 3.0, 40%) as label noise increased [24]. The approach taken in the present study differs in that it introduces systematic label noise using noise at random [25], increasing both the FP and FN rates linearly. Introducing noise into the training set resulted in a gradual decline in model performance, with both ROC AUC and AP scores decreasing as the level of noise increased. The model was particularly sensitive to FP, which caused a more pronounced decline in performance compared to FN. Introducing noise into the test set also impacted model performance, but the effects were more complex. The ROC AUC consistently decreased as FP rates increased, indicating that the model’s ability to distinguish between classes was compromised. However, the AP score showed a different pattern, with an initial decline followed by an increase as noise levels were increased. The introduction of FN in the test set had a less pronounced effect on performance compared to FP, unless FP and FN were combined, which led to a more marked decline in the model’s overall performance.

The counterintuitive increase in the AP score as the FP rate in the test set increased can be attributed to the method of calculating AP. AP evaluates the precision-recall trade-off across different thresholds, specifically calculating the proportion of TP to the sum of (TP + FP). When most of the negative class in the test set is artificially converted to positive, the opportunity for FP to occur is significantly reduced. This reduction in potential FP leads to an increase in precision, which in turn increases the AP score. Additionally, this manipulation dramatically alters the (eg, class balance from 90% negative to 90% positive), further influencing the precision-recall dynamics and contributing to the observed ostensible increase in AP. In practical terms, this finding emphasizes that certain performance metrics such as AP can behave unexpectedly in the presence of extensive label noise or class imbalance, underscoring the importance of using multiple evaluation metrics that are robust to changes in classes (discrimination and calibration) to fully understand model performance. These results reinforce that deploying predictive models trained on unvalidated EHR data can amplify false-positive and false-negative risks.

This study has several limitations that may affect the generalizability of these findings. First, the analysis was conducted using data from a single hospital and did not perform any external validation with data from other hospitals or a formal temporal validation using a future period. Therefore, it is uncertain whether the findings would directly generalize to different clinical settings, particularly those with different screening practices, disease encoding, and EHR systems. Second, the CTD, which is treated as the ground truth, is manually maintained by the clinical team. While it is likely more accurate than the EHR, it is not immune to possible human errors or omissions. Any such errors in the CTD would affect the data validation results by erroneously labeling some EHR entries as false positives or false negatives. However, this should minimally impact ML modeling as only data that had agreement across both databases were included. Third, there is potential for model overfitting due to the lack of an external validation set and default parameters, which we attempted to mitigate with the use of k-fold cross-validation, a relatively simple linear model, and a hold-out test set. Finally, noise at random linearly increases the FP and FN rate in the dataset, which may not accurately reflect how errors in EHRs typically occur.

In conclusion, the identified discrepancies in EHR-recorded GDM diagnoses compared to “true” GDM diagnoses reflect broader concerns about the accuracy of EHRs for public health and ML applications. Further, the magnitude of inaccuracies may play an important role in maximizing the utility of EHRs in enhancing health care outcomes, particularly for conditions such as GDM. However, when these discrepancies remain a small percentage (eg, <5%) of the dataset, such as in the case of this study, there was no noticeable impact on model training performance. Conversely, the risk of incorrect model evaluation increases when the test set labels are impacted by noise, as this has a more pronounced effect on performance metrics. These observations emphasize the importance of incorporating robust data cleaning, preprocessing, and validation methodologies in the development of ML models for health care. Future efforts should aim at developing standardized validation protocols for EHRs to ensure high data quality for training and evaluating ML algorithms. Such protocols could include harmonizing how GDM diagnoses are recorded across different sites, implementing automated consistency checks (for instance, prompting for a GDM diagnosis entry in the EHR when a laboratory result confirming GDM is received), and performing regular audits comparing EHR records with reference databases or laboratory results. By improving the integrity of data entry and maintenance in EHR systems, these measures could reduce discrepancies and enhance the utility of EHR data for both clinical care and ML applications.

## Abbreviations:

AP: average precision
CTD: clinical team database
CTD-GDM: gestational diabetes mellitus label as recorded in the clinical team database (reference standard label)
EHR: electronic health record
EHR-GDM: gestational diabetes mellitus label as recorded in the electronic health record
FN: false negative
FP: false positive
GDM: gestational diabetes mellitus
IADPSG: International Association of the Diabetes and Pregnancy Study Groups
*ICD*: *International Statistical Classification of Diseases and Related Health Problems*
LR: logistic regression
ML: machine learning
ROC AUC: area under the receiver operating characteristic curve
SNOMED: Systematized Nomenclature of Medicine
TN: true negative
TP: true positive
VAL-GDM: validated gestational diabetes mellitus label (only in cases where electronic health record and clinical team database labels matched)
XGBoost: Extreme Gradient Boosting
